# Influence of the Number of Channels and Classification Algorithm on the Performance Robustness to Electrode Shift in Steady-State Visual Evoked Potential-Based Brain-Computer Interfaces

**DOI:** 10.3389/fninf.2021.750839

**Published:** 2021-10-22

**Authors:** Hodam Kim, Chang-Hwan Im

**Affiliations:** ^1^Department of Biomedical Engineering, Hanyang University, Seoul, South Korea; ^2^Department of Electronic Engineering, Hanyang University, Seoul, South Korea; ^3^Department of HY-KIST Bioconvergence, Hanyang University, Seoul, South Korea; ^4^Department of Artificial Intelligence, Hanyang University, Seoul, South Korea

**Keywords:** brain-computer interface (BCI), steady-state visual evoked potential (SSVEP), performance robustness, classification algorithm, electrode configurations

## Abstract

There remains an active investigation on elevating the classification accuracy and information transfer rate of brain-computer interfaces based on steady-state visual evoked potential. However, it has often been ignored that the performance of steady-state visual evoked potential (SSVEP)-based brain-computer interfaces (BCIs) can be affected through the minor displacement of the electrodes from their optimal locations in practical applications because of the mislocation of electrodes and/or concurrent use of electroencephalography (EEG) devices with external devices, such as virtual reality headsets. In this study, we evaluated the performance robustness of SSVEP-based BCIs with respect to the changes in electrode locations for various channel configurations and classification algorithms. Our experiments involved 21 participants, where EEG signals were recorded from the scalp electrodes densely attached to the occipital area of the participants. The classification accuracies for all the possible cases of electrode location shifts for various channel configurations (1–3 channels) were calculated using five training-free SSVEP classification algorithms, i.e., the canonical correlation analysis (CCA), extended CCA, filter bank CCA, multivariate synchronization index (MSI), and extended MSI (EMSI). Then, the performances of the BCIs were evaluated using two measures, i.e., the average classification accuracy (ACA) across the electrode shifts and robustness to the electrode shift (RES). Our results showed that the ACA increased with an increase in the number of channels regardless of the algorithm. However, the RES was enhanced with an increase in the number of channels only when MSI and EMSI were employed. While both ACA and RES values for the five algorithms were similar under the single-channel condition, both ACA and RES values for MSI and EMSI were higher than those of the other algorithms under the multichannel (i.e., two or three electrodes) conditions. In addition, EMSI outperformed MSI when comparing the ACA and RES values under the multichannel conditions. In conclusion, our results suggested that the use of multichannel configuration and employment of EMSI could make the performance of SSVEP-based BCIs more robust to the electrode shift from the optimal locations.

## Introduction

The brain-computer interface (BCI) allows the users to communicate with the external world using their brain activities without standard communication methods, such as speaking and through gestures (Wolpaw et al., 2002). Various recording modalities have been used to implement BCIs, such as electroencephalography (EEG) (Lotte et al., 2018), magnetoencephalography (Mellinger et al., 2007), near-infrared spectroscopy (Hwang et al., 2014, 2016), electrocorticography (Hill et al., 2006; Schalk et al., 2007), and local field potential (Asgharpour et al., 2021). Among these modalities, EEG has been the most widely used owing to its advantages, such as non-invasiveness, excellent temporal resolution, convenience of use, and cost-effectiveness (Han et al., 2013; Hwang et al., 2013; Oikonomou et al., 2016; Lotte et al., 2018).

Electroencephalography-based BCIs convert electrical potentials measured on the scalp of the user while they perform a specific mental task, into designated commands. These commands are applied to various applications such as mental spellers (Hwang et al., 2012; Lim et al., 2015; Speier et al., 2017), assistive technology for patients (Hwang et al., 2017; Lim et al., 2017), and online home appliance control (Park et al., 2020). EEG-based BCIs can be classified based on the paradigms to elicit neural activities (Hwang et al., 2013) as motor imagery (MI)-based BCI (Acqualagna et al., 2016; León et al., 2020), visual P300-based BCI (Gu et al., 2019), steady-state visual evoked potential (SSVEP)-based BCI, non-motor mental imagery-based BCI (Kristensen et al., 2020), auditory BCI (Kim et al., 2011; Simon et al., 2015), and hybrid BCI (Jalilpour et al., 2020). Among these, SSVEP-based BCIs have the advantage of higher accuracy and higher information transfer rate (ITR) and generally require no/short training time (Tello et al., 2014; Nakanishi et al., 2015; Hwang et al., 2017; Xing et al., 2018).

Steady-state visual evoked potential-based BCIs recognize the intentions of the users by identifying the frequency of the visual stimulus. SSVEP is a quasi-periodic brain signal elicited through visual stimulus flickering or reversing at a specific frequency, generally recorded in the occipital region of the brain (Vialatte et al., 2010; Xu et al., 2014). Power spectral density analysis has traditionally been used to classify the frequency of SSVEP (Cheng et al., 2002; Cao et al., 2015). Recently, various algorithms have been proposed to enhance the classification accuracy and ITR of SSVEP-based BCIs, such as canonical correlation analysis (CCA) (Lin et al., 2007), least absolute shrinkage and selection operator (Zhang et al., 2012), multivariate synchronization index (MSI) (Zhang et al., 2014), filter bank CCA (FBCCA) (Chen et al., 2015), and task-related component analysis (Nakanishi et al., 2018).

Although elevating the classification accuracy and ITR of SSVEP-based BCIs is important, maintaining consistently high performance in the BCIs is an equally important factor to be considered for long-term and/or daily-life BCI applications. One factor that degrades the consistency of performance in the SSVEP-based BCI is the electrode dislocation from the optimal electrode locations, e.g., O1, O2, and Oz locations were generally used for a three-electrode BCI. It is generally not possible to reattach the electrodes to an identical location on the scalp for every usage in a BCI system. In addition, the locations of the electrodes can be slightly modified when the EEG device is concurrently used with external devices, such as a virtual reality head-mounted display (HMD) headset. However, to the best of our knowledge, the influence of electrode shift on the performance of SSVEP-based BCIs has not yet been quantitatively evaluated.

Park et al. (2013) evaluated feature extraction methods for MI-based BCIs in terms of performance robustness to slight changes in the electrode location and recommended the cross-correlation method as a promising feature extraction method. Except for this study, the robustness of performance against the electrode location shift has not been studied for EEG-based BCIs. On the contrary, the robustness of performance to the electrode shift was mainly investigated for electromyography (EMG)-based myoelectric interfaces. Boschmann and Platzner (2012) showed that the degradation of pattern recognition performance due to electrode shift could be improved by increasing the number of electrode channels and expanding the recording locations. Pan et al. (2015) demonstrated that the performance robustness against electrode shift in myoelectric prostheses based on high-density EMG could be improved by employing the multiclass common spatial pattern feature. Fan et al. (2016) proposed a novel CCA-based method to eliminate the reduction in performance in a surface EMG-based motion classification system. The recent work of Lv et al. (2018) proposed a novel neural network model using an autoencoder to extract the robust features against electrode shift.

With the recent growing interest in the practical use of SSVEP-based BCIs, there is a growing need for studies evaluating the performance robustness under various conditions, such as the number of channels and classification algorithms. This study aimed to investigate the performance variation in SSVEP-based BCIs owing to minor electrode shifts and to discover the conditions that minimize the variations in classification accuracy while maintaining high classification accuracy. The stimulus frequencies used for SSVEP-based BCIs can be classified into three frequency bands: low (~12 Hz), medium (12–30 Hz), and high (>30 Hz) frequency bands (Regan, 1989). The majority of SSVEP-based BCI studies were based on low-frequency and medium-frequency bands, since SSVEP responses are stronger in low-frequency bands than in high-frequency bands (Zhu et al., 2010; Liang et al., 2019). Therefore, in this study, we primarily investigated the performance robustness of the low-frequency SSVEP-based BCIs. To this aim, an experiment eliciting the SSVEP was conducted with 21 healthy participants and the EEG signals were recorded from the electrodes densely attached to their occipital area. Then, the performance robustness of SSVEP-based BCIs to the electrode shift with respect to the numbers of channels and classification algorithms was quantitatively investigated.

## Materials and Methods

### Participants

In this study, 25 healthy volunteers (15 men and 10 women; age 23.52 ± 2.73 years) with normal or corrected-to-normal vision had participated. None of them had any history of neurological, psychiatric, or other severe diseases that could affect the experimental results. The participants signed an informed consent form and were familiarized with the detailed protocol of the experiment. After completion of the experiment, each participant received a monetary reimbursement. Data from four participants were excluded from further analyses because of the non-existence of spectral peaks. The remaining data (14 men and 7 women; age 23.9 ± 2.79 years) were used in this study. This experimental study was approved and reviewed by the Institutional Review Board (IRB) of Hanyang University, Korea (HYI-14-167-11).

### Experiments and Data Acquisition

**Figure 1:**
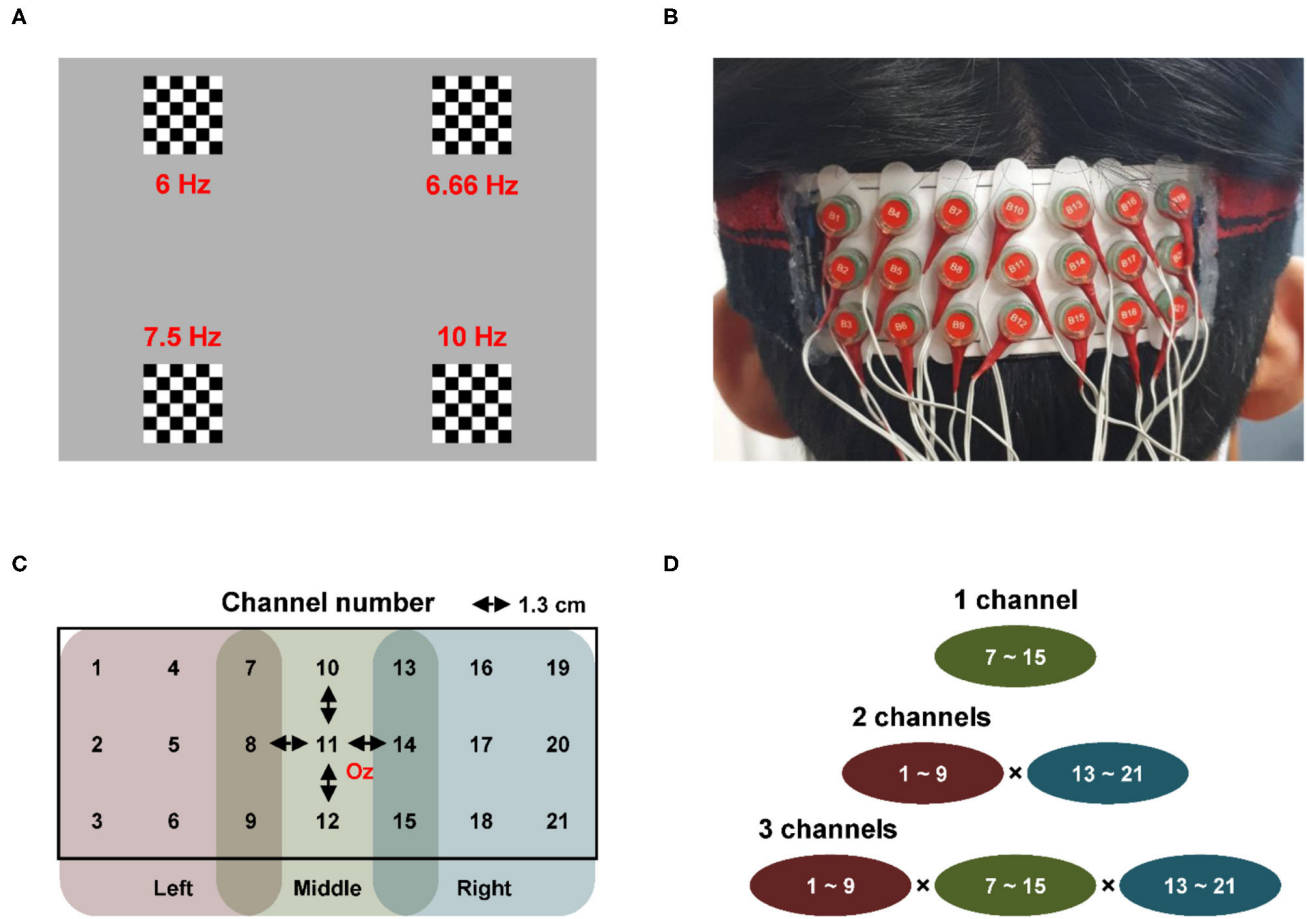
Experiment setup and channel combination **(A)** four 6 × 6 pattern-reversal checkerboard stimuli [frequencies of the four stimuli were set to 6 Hz (top left), 6.66 Hz (top right), 7.5 Hz (bottom left), and 10 Hz (bottom right)] **(B)** fabricated electrode array pad with dense electrode configuration **(C)** array pad [distance between the two neighboring electrodes was set to 1.3 cm,; center of the array pad was placed at Oz and according to the extended international 10–20 system; 21 channels were categorized into three categories depending on the location of the channel: left (channel 1–9), middle (channel 7–15), and right (channel 13–21)] **(D)** possible channel combinations with respect to the number of channels used for classification [for example, when two EEG signals recorded at two channels were used to classify the frequency of SSVEP, the classification accuracy was calculated in a total 81 channel combinations by combining the left 9 channels (channel 1–9) and right 9 channels (channel 13–21)].

Four 6 × 6 pattern-reversal checkerboard stimuli reversing at different frequencies were used to elicit the SSVEP responses. The four frequencies were set to 6 Hz (top left), 6.66 Hz (top right), 7.5 Hz (bottom left), and 10 Hz (bottom right), considering the intrinsic monitor refreshing rate. Figure 1A illustrates the configuration of the visual stimuli. In each trial, four visual stimuli were presented for 5 s with a 2 s inter-stimulus interval. The participant gazed at one of the four stimuli without blinking the eyes and making no body movements according to the instructions provided immediately before the stimulus presentation. One session consisted of 20 trials, and five sessions were conducted for each participant with a variable time interval between the sessions. Note that the participants could take a rest as long as they wanted during the inter-session intervals for reducing visual fatigue. The order of stimulus presentation was randomized and counterbalanced to avoid the potential habituation effects. The visual stimuli were displayed on a 24-in LCD monitor screen with a 60 Hz refresh rate, placed approximately 70 cm away from the eyes of the participant. The visual stimuli were generated and controlled using the Cogent-2000 toolbox (www.vislab.ucl.ac.uk/cogent.php; Wellcome Trust Centre for Neuroimaging and Institute of Cognitive Neuroscience, UCL, London).

Electroencephalography signals were recorded using a commercial biosignal recording system (ActiveTwo; Biosemi, Amsterdam, the Netherlands) from the 21 electrodes at a sampling rate of 2,048 Hz. A lab-made electrode pad was used to record the EEG signals from the 21 electrodes attached to the occipital area of the participants. Figure 1B shows the fabricated electrode array pad with a sports headband that mounts the electrodes densely on the occipital area. Figure 1C shows the detailed configuration of the electrodes on the pad, which were arranged in three rows and seven columns, and the distance between two neighboring electrodes was set to be 1.3 cm. EEG electrodes used for measuring the SSVEPs are usually attached to O1, Oz, and O2 according to the extended international 10–20 system (Meng et al., 2011; Zhang et al., 2012; Lim et al., 2015, 2017; Hwang et al., 2017). Therefore, the center of the array pad (Channel-11) was placed at Oz, when Channel-5 and Channel-17 corresponded to O1 and O2, respectively. A common mode sense active electrode and a driven right leg passive electrode were attached to the left and right mastoids, respectively, to form a feedback loop for the amplifier reference (www.biosemi.com/faq/cms&drl.htm).

### Analysis

All analyses, including the signal preprocessing, classification, and statistical analysis, were performed using MATLAB R2019b (Mathworks, Natick, MA, USA).

#### Preprocessing

Recorded EEG signals were downsampled at 256 Hz considering the computational efficiency and were then band-pass filtered using a third-order zero-phase Butterworth filter with cutoff frequencies of 4 and 52 Hz. We investigated the SSVEP responses elicited by flickering stimuli using EEG signals recorded from Channels 5, 11, and 17. Please note that we excluded the data from four participants whose SSVEP responses were not observed, as mentioned in section Participants. Supplementary Figure 1 shows the average amplitude spectra averaged across 21 subjects and 25 trials for each stimuli frequency at channels O1, Oz, and O2, where clear SSVEP peaks were observed at the target frequencies (6, 6.66, 7.5, and 10, which corresponds to 60/10, 60/9, 60/8, and 60/6) and their harmonics. For each trial, the visual stimulus that the participant was staring at was identified using five different classification algorithms: CCA, extended CCA (ECCA), FBCCA, MSI, and extended MSI (EMSI) (Zhang et al., 2017). The preprocessed EEG datasets are available at the following link: https://figshare.com/s/cd46e1531d67f3023fa6.

#### Classification Algorithms

Canonical correlation analysis is a multivariate statistical method used to determine the linear combinations, which maximize the correlation between the two data sets *X* and *Y* (Lin et al., 2007; Bin et al., 2009). The weight vectors *W*_*X*_ and *W*_*Y*_ that maximize the canonical correlation between ${x=X^{T}W}_{X}$ and ${y=Y^{T}W}_{Y}$ were determined by solving the following optimization problem:


(1) $$\begin{array}{l} \underset{W_{x},W_{y}}{max}\rho \left(x,y\right)=\frac{W_{X}^{T}XY^{T}W_{Y}}{\sqrt{W_{X}^{T}XX^{T}W_{X}\cdot W_{Y}^{T}YY^{T}W_{Y}}}, \end{array}$$

where ρ is the canonical correlation coefficient. In the SSVEP frequency classification, *X* and *Y* are the EEG signals and SSVEP reference signals, respectively. The reference signals for each stimulus frequency were computed as follows:


(2) $$\begin{array}{l} Y=\left[\begin{array}{c} \sin\left(2\pi f\frac{t}{F_{s}}\right)\\ \cos\left(2\pi f\frac{t}{F_{s}}\right)\\ \vdots \\ \sin\left(2\pi N_{h}f\frac{t}{F_{s}}\right)\\ \cos\left(2\pi N_{h}f\frac{t}{F_{s}}\right) \end{array}\right],\;t=1,\;2,\cdots \;,M\;, \end{array}$$

where *N*_*h*_ is the number of harmonics, *F*_*s*_ is the sampling rate, *M* is the number of signal samples, and *f* is the base frequency of each stimulus frequency. The number of harmonics for the five classification algorithms was set to four for achieving the highest average classification accuracy for the optimal electrode positions. The canonical correlation coefficients were calculated for all the stimulus frequencies. Then, the frequency of the largest coefficient was selected.

Multivariate synchronization index measures the synchronization between the two datasets, similar to the coefficient of CCA for classifying the stimulus frequency (Zhang et al., 2014). Let *X* and *Y* denote the EEG and reference signals, respectively. The size of matrix *X* is *N* × *M* and the size of *Y* is 2*N*_*h*_ × *M*. Here, *N* is the number of channels in the EEG signals, *N*_*h*_ is the number of harmonics, and *M* is the number of samples. The correlation matrix of *X* and *Y* was calculated as follows:


(3) $$\begin{array}{l} C=\;\left[\begin{array}{cc} C_{11} & C_{12}\\ C_{21} & C_{22} \end{array}\right], \end{array}$$

where $C_{11}=\frac{1}{M}XX^{T}$, $C_{22}=\frac{1}{M}YY^{T}$, $C_{12}=C_{21}=\frac{1}{M}XY^{T}$, and *M* is the number of samples. Because the synchronization measure can be influenced by autocorrelation, the following linear transformation was applied to matrix *C* (Joudaki et al., 2012).


(4) $$\begin{array}{l} U=\;\left[\begin{array}{cc} {C_{11}}^{-\frac{1}{2}} & 0\\ 0 & {C_{22}}^{-\frac{1}{2}} \end{array}\right] \end{array}$$

The transformed matrix was given by


(5) $$\begin{array}{l} R=UCU^{T}, \end{array}$$

where the dimension of *R* is *N* + 2*N*_*h*_. Let λ_1_, λ_2_, ⋯ , λ_*P*_, where *P* = *N*+2*N*_*h*_, be the Eigenvalues of matrix *R*. The Eigenvalues were normalized as follows: $\lambda_{i}^{{\;}^{\prime }}=\frac{\lambda_{i}}{\overset{P}{\underset{i=1}{\sum }}\lambda_{i}}$. Then, the synchronization index between *X* and *Y* was calculated as follows:


(6) $$\begin{array}{l} S=1+\frac{\overset{P}{\underset{i=1}{\sum }}\lambda_{i}^{{\;}^{\prime }}\log\lambda_{i}^{{\;}^{\prime }}}{\log P} \end{array}$$

The synchronization indices were calculated for all the stimulus frequencies and the frequency of the largest synchronization index was selected.

Extended MSI is an algorithm that extends the MSI by incorporating time-delayed EEG signals during the calculation of the synchronization index (Zhang et al., 2017). ECCA is an algorithm that extends the CCA similar to the EMSI. Let *X* be the EEG signal. The delayed signals, *X*^τ^, were appended to *X* as


(7) $$\begin{array}{l} \bar{X}=\left[\begin{array}{c} X\\ X^{\tau } \end{array}\right], \end{array}$$

where τ is the number of delayed samples (Zhang et al., 2017). In this study, we generated the delayed signals using the circular shift method that moves the final sample of a signal to the first position while shifting all the other samples of the signal to the next position. We shifted the circular samples of the EEG signals once, i.e., τ was set to 1. Then, the new synchronization index and the new canonical correlation coefficient using $\bar{X}$ were calculated using Equations (2)–(6) and (1)–(2), respectively. The target frequency was then estimated in a manner similar to the MSI and CCA.

Filter bank CCA decomposes the EEG signals into multiple sub-band components and calculates the canonical correlation coefficient of each sub-band component. The weighted sum of the squared CCA coefficients corresponding to all the sub-band components was calculated as follows:


(8) $$\begin{array}{l} \rho_{f}=\overset{N_{sb}}{\underset{n=1}{\sum }}w\left(n\right)\cdot {\left(\rho_{f}^{n}\right)}^{2}, \end{array}$$

where *N*_*sb*_ is the number of sub-bands and *n* is the index of the sub-band. The weights of the sub-band components were defined as follows:


(9) $$\begin{array}{l} w\left(n\right)=n^{-a}+b, \end{array}$$

where *a* and *b* are constants. In this study, three parameters were determined based on the previous studies (Chen et al., 2015) (*a* = 1.25, *b* = 0.25, and *N*_*sb*_ = 5). The lower and upper cutoff frequencies of the five sub-bands were designed as follows: 4–52 Hz, 8–52 Hz, 12–52 Hz, 16–52 Hz, and 20–52 Hz. The weighted sum of the squared CCA coefficients (ρ_*f*_) was calculated for all the stimulus frequencies. The frequency with the maximum ρ_*f*_ was then selected as the target frequency.

#### Performance Evaluation Measures

Classification accuracies were calculated for five training-free SSVEP classification algorithms for all the possible electrode shift cases with respect to different numbers of channels (1–3 channels). As shown in Figures 1C, 21 channels were divided into three-channel groups: the left 9 channels (Channels 1–9), middle 9 channels (Channels 7–15), and right 9 channels (Channels 13–21). In each channel group, the central channels (left: Channel 5, middle: Channel 11, right: Channel 17) corresponded to the original channel positions (O1, Oz, and O2, respectively), and the remaining eight channels in each group corresponded to the position where the electrodes could be shifted from their original positions.

There can be various channel combinations when the number of channels is 1, 2, and 3, as illustrated in Figure 1D. When the number of channels was 1, the frequency of SSVEP was classified using only one EEG signal recorded from one channel. In this case, the classification accuracies were calculated for each of the middle nine channels (Channels 7–15). When two EEG signals recorded at two different channels were used for the classification, the classification accuracies were calculated for each of 81 channel combinations by combining the left nine channels (Channels 1–9) and right nine channels (Channels 13–21). Finally, when the number of channels was 3, classification accuracies were calculated for each of 675 channel combinations by combining the left 9 channels, middle 9 channels, and right 9 channels. In the combination of these three groups, the combinations with specific channels that overlapped with each other were excluded, e.g., Channels 7, 7, and 13.

To compare the changes in the classification performance owing to the electrode shift, two measures were calculated for each subject: (1) average classification accuracy across the electrode shift (ACA) and (2) robustness to the electrode shift (RES). The ACA was calculated by averaging the classification accuracies for all the channel combinations. The RES was evaluated by calculating the coefficient of variation (CV) of the classification accuracies for all the channel combinations, with the RES value being defined as 1 – CV. CV is a standardized measure of dispersion, which is defined as the ratio of the standard deviation to the mean. A low-RES value indicates that the classification performance is affected significantly by the changes in the electrode locations, whereas a high-RES value indicates that the classification performance is not affected significantly by the electrode shift.

#### Statistical Analysis

A two-way analysis of variance with repeated measures based on two factors (the number of channels and the classification algorithm) was used to statistically compare the ACA and RES. A paired *t*-test with Bonferroni correction was used for the *post-hoc* analyses.

## Results

### Statistical Comparison

First, we statistically compared the ACA and RES values with respect to the number of channels and classification algorithms for window lengths of 2–5 s with intervals of 0.5 s. In Table 1, the two-way repeated-measures ANOVA for ACA showed a statistically significant main effect on the number of channels and the algorithms for all the window lengths. The interaction between the number of channels and the algorithm was statistically significant for each window length. In contrast, the two-way repeated-measures ANOVA for RES showed a statistically significant main effect only for the classification algorithms for every window length, when the interaction between the number of channels and the algorithm was statistically significant for each window length. While the main effect of the number of channels on the RES value was not significant, we statistically compared the ACA and RES of the three levels of the number of channels and five levels of algorithms using a paired *t*-test as a *post-hoc* analysis because the interaction of ACA and RES was found to be statistically significant for every window length.

**Table 1:** Results of the two-way repeated-measures ANOVA with two factors of “Number of channels” and “Algorithm” for various window lengths.

**Source (df, df error)**	**Window length (s)**						
	**2**	**2.5**	**3**	**3.5**	**4**	**4.5**	**5**
**Average classification accuracy across the electrode shift (ACA)**							
Number of channels (2, 40)	10.681***	9.454***	10.435***	12.954***	13.305***	16.966***	19.156***
Algorithm (4, 80)	4.440**	4.532**	5.670***	4.954**	5.440**	4.987**	4.772**
Number of channels × Algorithm (8, 160)	6.659***	10.261***	10.754***	8.820***	8.657***	7.309***	5.076***
**Robustness to the electrode shift (RES)**							
Number of channels (2, 40)	n.s.	n.s.	n.s.	n.s.	n.s.	n.s.	n.s.
Algorithm (4, 80)	17.865***	24.256***	24.904***	24.337***	18.062***	15.599***	12.433***
Number of channels × Algorithm (8, 160)	17.385***	17.360***	24.962***	17.123***	20.358***	17.102***	14.162***

*Values denote the F-value (p-value); n.s. indicates the non-significant (p ≥ 0.05).*

Asterisks indicate the statistical significance (**p < 0.01, and ***p < 0.001).

### Average Classification Accuracy Across the Electrode Shift

**Figure 2:**
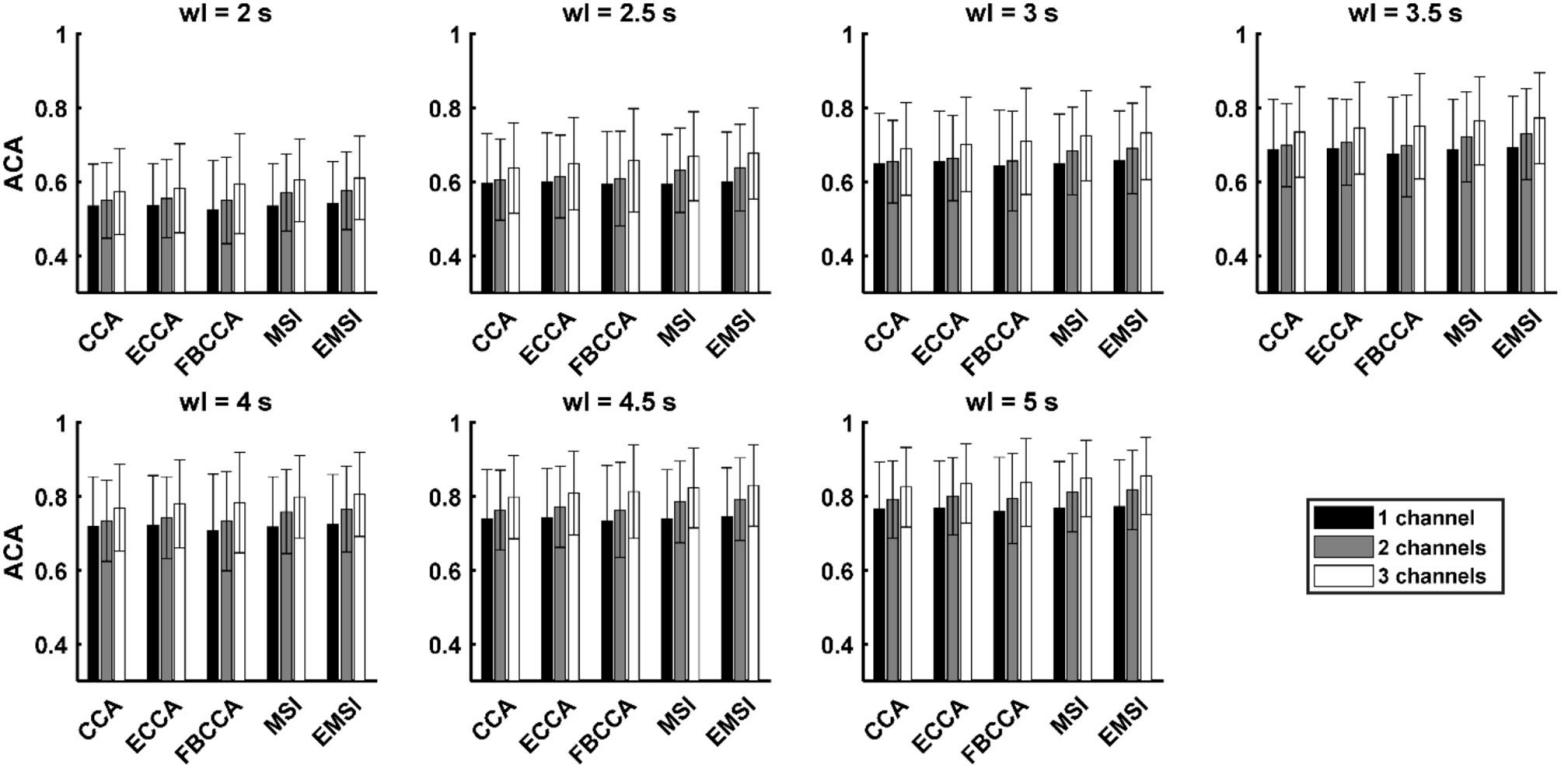
Mean and SD of the average classification accuracy across the electrode shift (ACA) across the subjects with five SSVEP classification algorithms in the case of 3 channels and seven window lengths (wl) (black, gray, and white bars denote the mean accuracies of 1 channel, 2 channels, and 3 channels, respectively; the error bars represent the SD ACA).

Figure 2 and Supplementary Table 1 show the average and SD of the ACA across the 21 subjects. Table 2 shows the results of the paired *t*-test for the number of channels with respect to the algorithm and window length. When classifying the frequency of SSVEP using the CCA, ECCA, and FBCCA, there was no statistically significant difference between the ACA for one channel and that for two channels in each window length. However, the ACA for three channels was significantly higher than those for one channel and two channels (CCA: 4.5 and 5 s; ECCA: 3.5, 4, 4.5, and 5 s; FBCCA: all window lengths). When the frequency of SSVEP was classified using MSI and EMSI, the ACA was significantly increased with an increase in the number of channels in every window length. In short, the ACA for three channels was higher than that for one and two channels regardless of the algorithms. The ACA for two channels increased significantly compared to that for one channel in MSI and EMSI.

**Table 2:** Bonferroni corrected *p*-values of *post-hoc* analysis among the average classification accuracies across the electrode shift (ACAs) of three different numbers of channels for classification algorithm and window length.

**Algorithm**	**Number of channels (left vs. right)**	**Window length (s)**						
		**2**	**2.5**	**3**	**3.5**	**4**	**4.5**	**5**
CCA	1 vs. 2	n.s.	n.s.	n.s.	n.s.	n.s.	n.s.	n.s.
	1 vs. 3	n.s.	n.s.	n.s.	n.s.	n.s.	0.014	0.008
	2 vs. 3	0.011	0.001	0.000	0.000	0.000	0.000	0.000
ECCA	1 vs. 2	n.s.	n.s.	n.s.	n.s.	n.s.	n.s.	n.s.
	1 vs. 3	n.s.	n.s.	n.s.	0.035	0.025	0.008	0.003
	2 vs. 3	0.004	0.000	0.000	0.000	0.000	0.000	0.000
FBCCA	1 vs. 2	n.s.	n.s.	n.s.	n.s.	n.s.	n.s.	n.s.
	1 vs. 3	0.002	0.003	0.004	0.002	0.002	0.001	0.001
	2 vs. 3	0.000	0.000	0.000	0.000	0.000	0.000	0.000
MSI	1 vs. 2	0.019	0.047	0.041	0.030	0.014	0.003	0.003
	1 vs. 3	0.000	0.001	0.000	0.000	0.000	0.000	0.000
	2 vs. 3	0.000	0.000	0.000	0.000	0.000	0.000	0.000
EMSI	1 vs. 2	0.023	0.030	0.036	0.020	0.009	0.002	0.001
	1 vs. 3	0.000	0.000	0.000	0.000	0.000	0.000	0.000
	2 vs. 3	0.000	0.000	0.000	0.000	0.000	0.000	0.000

*n.s. indicates the non-significant (Bonferroni corrected p > 0.05).*

*A positive value indicates that the ACA of the right is higher than that of the left*.

Table 3 shows the results of the paired *t*-test among the algorithms with respect to the number of channels and window lengths. In the case of one channel, the ACA of EMSI was significantly higher than that of CCA (window length: 4, 4.5, and 5 s) and MSI (window length: 2.5, 4, 4.5, and 5 s). The ACA of ECCA was significantly higher than that of MSI in the case with a window length of 2.5 s. In the case of two channels, the ACAs of ECCA, MSI, and EMSI were significantly higher than that of CCA (ECCA: every window length except 3.5 s; MSI and EMSI: every window length). The ACAs of MSI and EMSI were significantly higher than that of ECCA (MSI: 2.5, 3, and 4 s; EMSI: every window length). The ACA of EMSI was significantly higher than that of MSI (window length: 3.5, 4, 4.5, and 5 s). Finally, in the case of three channels, the ACAs of ECCA, MSI, and EMSI were significantly higher than that of CCA in each window length. The ACAs of MSI and EMSI were significantly higher than that of ECCA (MSI: 3 s; EMSI: each window length except 2 s). The ACA of EMSI was significantly higher than that of MSI (window length: 3.5, 4, 4.5, and 5 s). Taken together, ACAs of MSI and EMSI were significantly higher than that of CCA and ECCA in the multichannel condition. The ACA of EMSI was significantly higher than that of the other algorithms except for FBCCA regardless of the number of channels.

**Table 3:** Bonferroni corrected *p*-values of *post-hoc* analysis among the average classification accuracies across the electrode shift (ACA)s in five different algorithms for the number of channels and window length.

**Number of channels**	**Algorithm (left vs. right)**	**Window length (s)**						
		**2**	**2.5**	**3**	**3.5**	**4**	**4.5**	**5**
1	CCA vs. ECCA	n.s.	n.s.	n.s.	n.s.	n.s.	n.s.	n.s.
	CCA vs. FBCCA	n.s.	n.s.	n.s.	n.s.	n.s.	n.s.	n.s.
	CCA vs. MSI	n.s.	n.s.	n.s.	n.s.	n.s.	n.s.	n.s.
	CCA vs. EMSI	n.s.	n.s.	n.s.	n.s.	0.035	0.002	0.029
	ECCA vs. FBCCA	n.s.	n.s.	n.s.	n.s.	n.s.	n.s.	n.s.
	ECCA vs. MSI	n.s.	−0.045	n.s.	n.s.	n.s.	n.s.	n.s.
	ECCA vs. EMSI	n.s.	n.s.	n.s.	n.s.	n.s.	n.s.	n.s.
	FBCCA vs. MSI	n.s.	n.s.	n.s.	n.s.	n.s.	n.s.	n.s.
	FBCCA vs. EMSI	n.s.	n.s.	n.s.	n.s.	n.s.	n.s.	n.s.
	MSI vs. EMSI	n.s.	0.043	n.s.	n.s.	0.031	0.002	0.025
2	CCA vs. ECCA	0.037	0.002	0.001	n.s.	0.049	0.005	0.010
	CCA vs. FBCCA	n.s.	n.s.	n.s.	n.s.	n.s.	n.s.	n.s.
	CCA vs. MSI	0.012	0.003	0.002	0.022	0.003	0.002	0.000
	CCA vs. EMSI	0.009	0.002	0.001	0.008	0.001	0.000	0.000
	ECCA vs. FBCCA	n.s.	n.s.	n.s.	n.s.	n.s.	n.s.	n.s.
	ECCA vs. MSI	n.s.	0.042	0.034	n.s.	0.049	n.s.	n.s.
	ECCA vs. EMSI	0.038	0.016	0.012	0.037	0.006	0.014	0.013
	FBCCA vs. MSI	n.s.	n.s.	n.s.	n.s.	n.s.	n.s.	n.s.
	FBCCA vs. EMSI	n.s.	n.s.	n.s.	n.s.	n.s.	n.s.	n.s.
	MSI vs. EMSI	n.s.	n.s.	n.s.	0.004	0.009	0.003	0.001
3	CCA vs. ECCA	0.003	0.000	0.000	0.003	0.001	0.001	0.000
	CCA vs. FBCCA	n.s.	n.s.	n.s.	n.s.	n.s.	n.s.	n.s.
	CCA vs. MSI	0.020	0.005	0.002	0.008	0.005	0.008	0.002
	CCA vs. EMSI	0.009	0.004	0.002	0.004	0.002	0.001	0.000
	ECCA vs. FBCCA	n.s.	n.s.	n.s.	n.s.	n.s.	n.s.	n.s.
	ECCA vs. MSI	n.s.	n.s.	0.047	n.s.	n.s.	n.s.	n.s.
	ECCA vs. EMSI	n.s.	0.045	0.019	0.041	0.021	0.036	0.017
	FBCCA vs. MSI	n.s.	n.s.	n.s.	n.s.	n.s.	n.s.	n.s.
	FBCCA vs. EMSI	n.s.	n.s.	n.s.	n.s.	n.s.	n.s.	n.s.
	MSI vs. EMSI	n.s.	n.s.	n.s.	0.008	0.005	0.004	0.002

*n.s. indicates the non-significant (Bonferroni corrected p > 0.05).*

*A positive value indicates that the ACA of the right is higher than that of the left.*

*A negative value indicates that the ACA of the left is higher than that of the right*.

### Robustness to the Electrode Shift

**Figure 3:**
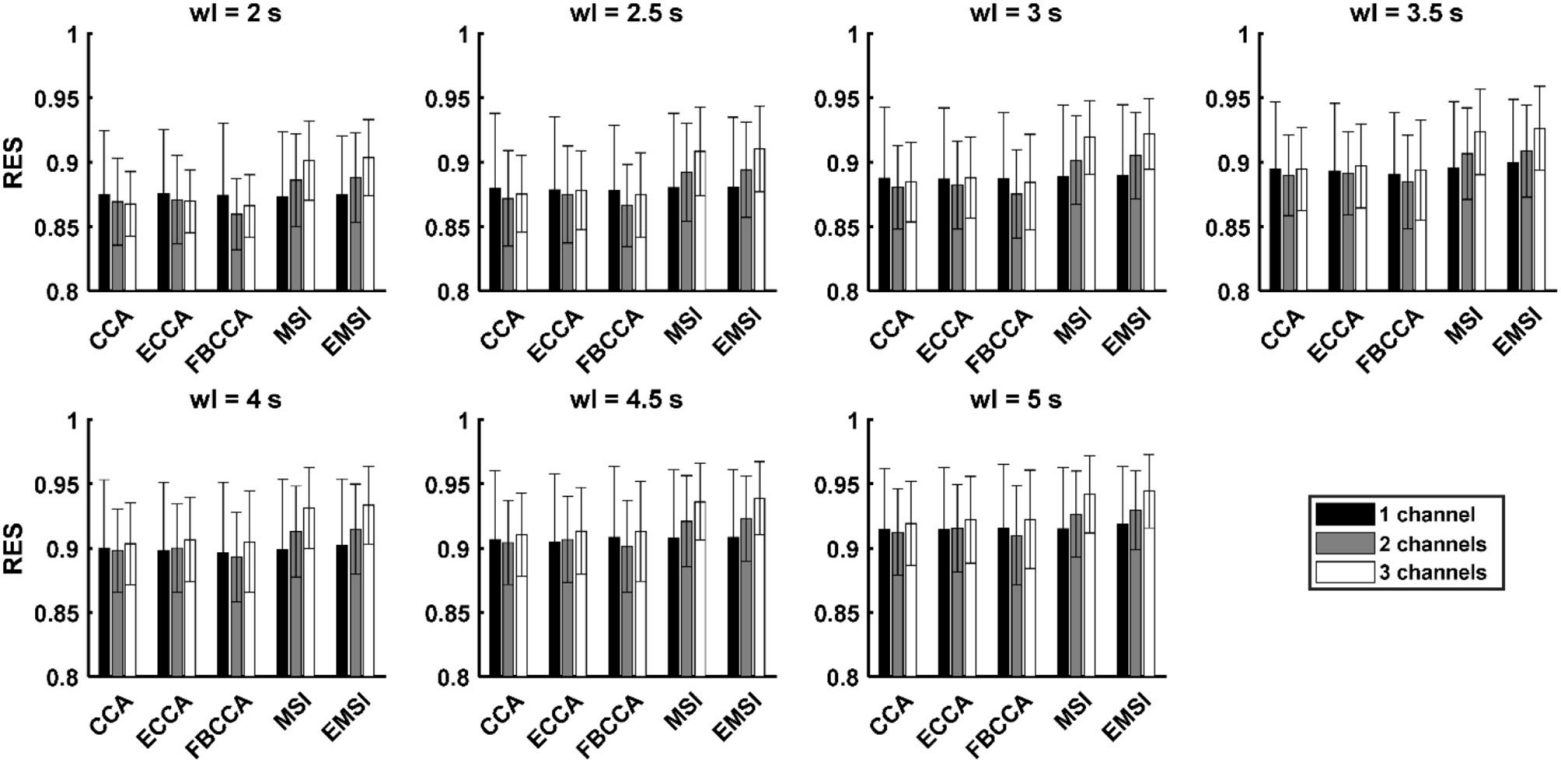
Mean and standard deviation of the robustness to the electrode shift (RES) across the subjects with five SSVEP classification algorithms in the case with the number of channels as 3 and window length (wl) as 7 (black, gray, and white bars denote the mean accuracies in the cases with the number of channels as 1, 2, and 3, respectively; the error bars represent standard deviations of RES).

Figure 3 and Supplementary Table 2 show the average and standard deviation of RES across the 21 subjects with respect to the number of channels and classification algorithm. Table 4 shows the results of the *post-hoc* analysis among the RESs in the case of three channels for each classification algorithm. There was no significant difference among the RESs of the three different numbers of channels for CCA and ECCA. In the case of FBCCA, the RES for three channels was significantly higher than that for two channels (window length: 4, 4.5, and 5 s). For MSI and EMSI, the RESs increased with an increase in the number of channels, and the RES for three channels was significantly higher than those for one channel and two channels in every window length. To sum up, the RES for three channels was higher than that for both one and two channels when MSI and EMSI were used.

**Table 4:** Bonferroni corrected *p*-values of *post-hoc* analysis among the robustness to the electrode shift (RES) in the cases with the number of channels as 1, 2, and 3 for classification algorithm and window length.

**Algorithm**	**Number of channels (left vs. right)**	**Window length (s)**						
		**2**	**2.5**	**3**	**3.5**	**4**	**4.5**	**5**
CCA	1 vs. 2	n.s.	n.s.	n.s.	n.s.	n.s.	n.s.	n.s.
	1 vs. 3	n.s.	n.s.	n.s.	n.s.	n.s.	n.s.	n.s.
	2 vs. 3	n.s.	n.s.	n.s.	n.s.	n.s.	n.s.	n.s.
ECCA	1 vs. 2	n.s.	n.s.	n.s.	n.s.	n.s.	n.s.	n.s.
	1 vs. 3	n.s.	n.s.	n.s.	n.s.	n.s.	n.s.	n.s.
	2 vs. 3	n.s.	n.s.	n.s.	n.s.	n.s.	n.s.	n.s.
FBCCA	1 vs. 2	n.s.	n.s.	n.s.	n.s.	n.s.	n.s.	n.s.
	1 vs. 3	n.s.	n.s.	n.s.	n.s.	n.s.	n.s.	n.s.
	2 vs. 3	n.s.	n.s.	n.s.	n.s.	0.023	0.007	0.001
MSI	1 vs. 2	n.s.	n.s.	n.s.	n.s.	n.s.	n.s.	n.s.
	1 vs. 3	0.004	0.043	0.018	0.015	0.007	0.013	0.011
	2 vs. 3	0.000	0.000	0.000	0.000	0.000	0.001	0.000
EMSI	1 vs. 2	n.s.	n.s.	n.s.	n.s.	n.s.	n.s.	n.s.
	1 vs. 3	0.002	0.013	0.008	0.013	0.006	0.008	0.009
	2 vs. 3	0.000	0.000	0.000	0.000	0.000	0.000	0.000

*n.s. indicates non-significant (Bonferroni corrected p > 0.05).*

*A positive value indicates that the RES of the right is higher than that of the left*.

Table 5 shows the results of the *post-hoc* analysis of the RESs of five classification algorithms for the number of channels. In the single-channel, there was no statistically significant difference among the RESs of the five algorithms. The RESs of MSI and EMSI were significantly higher than that of CCA, ECCA, and FBCCA for every window length when using multiple channels (two channels and three channels). In addition, in the case of three channels, the RES of ECCA was significantly higher than that of CCA (window length: 4 and 5 s), and the RES of EMSI was significantly higher than that of MSI (window length: 3.5, 4.5, and 5 s). In summary, the RESs of the five algorithms were similar under the single-channel condition. However, the RESs of MSI and EMSI were significantly higher than those of the other algorithms under multichannel conditions. In the case of three channels, the RES of EMSI was significantly higher than that of MSI.

**Table 5:** Bonferroni corrected *p*-values of *post-hoc* analysis among the robustness to electrode shift (RES) in four different algorithms for the number of channels and window lengths.

**Number of channels**	**Algorithm (left vs. right)**	**Window length (s)**						
		**2**	**2.5**	**3**	**3.5**	**4**	**4.5**	**5**
1	CCA vs. ECCA	n.s.	n.s.	n.s.	n.s.	n.s.	n.s.	n.s.
	CCA vs. FBCCA	n.s.	n.s.	n.s.	n.s.	n.s.	n.s.	n.s.
	CCA vs. MSI	n.s.	n.s.	n.s.	n.s.	n.s.	n.s.	n.s.
	CCA vs. EMSI	n.s.	n.s.	n.s.	n.s.	n.s.	n.s.	n.s.
	ECCA vs. FBCCA	n.s.	n.s.	n.s.	n.s.	n.s.	n.s.	n.s.
	ECCA vs. MSI	n.s.	n.s.	n.s.	n.s.	n.s.	n.s.	n.s.
	ECCA vs. EMSI	n.s.	n.s.	n.s.	n.s.	n.s.	n.s.	n.s.
	FBCCA vs. MSI	n.s.	n.s.	n.s.	n.s.	n.s.	n.s.	n.s.
	FBCCA vs. EMSI	n.s.	n.s.	n.s.	n.s.	n.s.	n.s.	n.s.
	MSI vs. EMSI	n.s.	n.s.	n.s.	n.s.	n.s.	n.s.	n.s.
2	CCA vs. ECCA	n.s.	n.s.	n.s.	n.s.	n.s.	n.s.	n.s.
	CCA vs. FBCCA	n.s.	n.s.	n.s.	n.s.	n.s.	n.s.	n.s.
	CCA vs. MSI	0.002	0.000	0.000	0.000	0.000	0.000	0.000
	CCA vs. EMSI	0.001	0.000	0.000	0.000	0.000	0.000	0.000
	ECCA vs. FBCCA	n.s.	n.s.	n.s.	n.s.	n.s.	n.s.	n.s.
	ECCA vs. MSI	0.003	0.000	0.000	0.000	0.000	0.000	0.000
	ECCA vs. EMSI	0.002	0.000	0.000	0.000	0.000	0.000	0.000
	FBCCA vs. MSI	0.000	0.000	0.000	0.000	0.001	0.004	0.016
	FBCCA vs. EMSI	0.000	0.000	0.000	0.000	0.000	0.001	0.002
	MSI vs. EMSI	n.s.	n.s.	n.s.	n.s.	n.s.	n.s.	n.s.
3	CCA vs. ECCA	n.s.	n.s.	n.s.	n.s.	0.022	n.s.	0.047
	CCA vs. FBCCA	n.s.	n.s.	n.s.	n.s.	n.s.	n.s.	n.s.
	CCA vs. MSI	0.000	0.000	0.000	0.000	0.000	0.000	0.000
	CCA vs. EMSI	0.000	0.000	0.000	0.000	0.000	0.000	0.000
	ECCA vs. FBCCA	n.s.	n.s.	n.s.	n.s.	n.s.	n.s.	n.s.
	ECCA vs. MSI	0.000	0.000	0.000	0.000	0.000	0.000	0.000
	ECCA vs. EMSI	0.000	0.000	0.000	0.000	0.000	0.000	0.000
	FBCCA vs. MSI	0.000	0.000	0.000	0.000	0.000	0.001	0.001
	FBCCA vs. EMSI	0.000	0.000	0.000	0.000	0.000	0.000	0.000
	MSI vs. EMSI	n.s.	n.s.	n.s.	0.007	n.s.	0.018	0.009

*n.s. indicates non-significant (Bonferroni corrected p > 0.05).*

*A positive value indicates that the RES of the right is higher than that of the left*.

### Optimal Configuration for the Robustness to the Electrode Shift

Comparisons of two measures, i.e., the ACA and RES, with respect to the number of channels showed that the ACA increased significantly regardless of the algorithms and the RES increased significantly when MSI and EMSI were used in the case of three channels. Comparisons of two measures with respect to the algorithms showed that the ACA and RES of MSI and EMSI were superior to those of the CCA and ECCA under multichannel conditions. In particular, in the case of three channels, the RES of EMSI was superior to that of the other four algorithms. Therefore, the optimal conditions that make the SSVEP-based BCI robust to the electrode shift were the multichannel configurations (especially three channels) and the employment of the EMSI algorithm.

## Discussion

The classification accuracy of SSVEP-based BCI is closely related to the signal-to-noise ratio (SNR) (Zhang et al., 2014; Kumar and Reddy, 2018). With an increase in the window length, the SNR of the SSVEP components increases, thereby leading to a higher classification accuracy (Xing et al., 2018). Similarly, as the number of channels increases, SNR of the SSVEP components can be enhanced by employing spatial filtering, also resulting in higher classification accuracy (Bin et al., 2009). Therefore, the variation in the accuracy owing to the electrode shift could decrease as the number of channels increases. In the MSI and EMSI, the S-estimator algorithm was used to measure the synchronization between the EEG signals and reference signals. Because the S-estimator is a non-linear measure of synchronization, both MSI and EMSI are known to have a lower probability of losing some useful information compared with CCA, ECCA, and FBCCA (Zhang et al., 2014; Zerafa et al., 2018). Additionally, the S-estimator has been proven robust to dynamic noises (Boccaletti et al., 2002; Carmeli et al., 2005; Jalili et al., 2007). Therefore, it is possible that MSI and EMSI based on S-estimator could classify the frequency of SSVEP more accurately at the surrounding positions of the original position compared with the other three algorithms.

Under the single-channel condition, the RES values of the five algorithms were similar as shown in Table 5. This result implies that the spatial filtering effect of five algorithms for strengthening the SSVEP components and suppressing the noises (Wong et al., 2020) was similar to each other in the case of a single channel. However, under the multichannel conditions, the RES values of MSI and EMSI were significantly higher than those of the other CCA-based algorithms.

Interestingly, we found that the variation in the classification performance due to the electrode shift increased as the number of channels increased when CCA and its variants (i.e., ECCA and FBCCA) were employed. As shown in Figures 2, 3, the ACA increased as the number of channels increased, but the RES for a single channel was higher than that for a multichannel. Particularly, this trend became more distinct for shorter window lengths with lower SNR. Bin et al. (2009) reported that the CCA weights from the parietal region and occipital region had the opposite sign, and thus the noise that these signals commonly have could be eliminated by employing the CCA weights. On the contrary, common noises may be enhanced if the CCA weights from different channels have the same sign. When two or three electrodes were shifted simultaneously, the SNR of SSVEP components could be either increased or decreased depending on the signs of the CCA weights, making the variation of the classification performance increased. As shown in Tables 2, 4, in the case of CCA-based algorithms, the ACA for three channels was significantly higher than those for single and two channels, while the RES did not increase as the number of channels increased. These results suggest that the CCA-based algorithms are not suitable for reducing the variation of classification performance due to the electrode dislocation.

**Figure 4:**
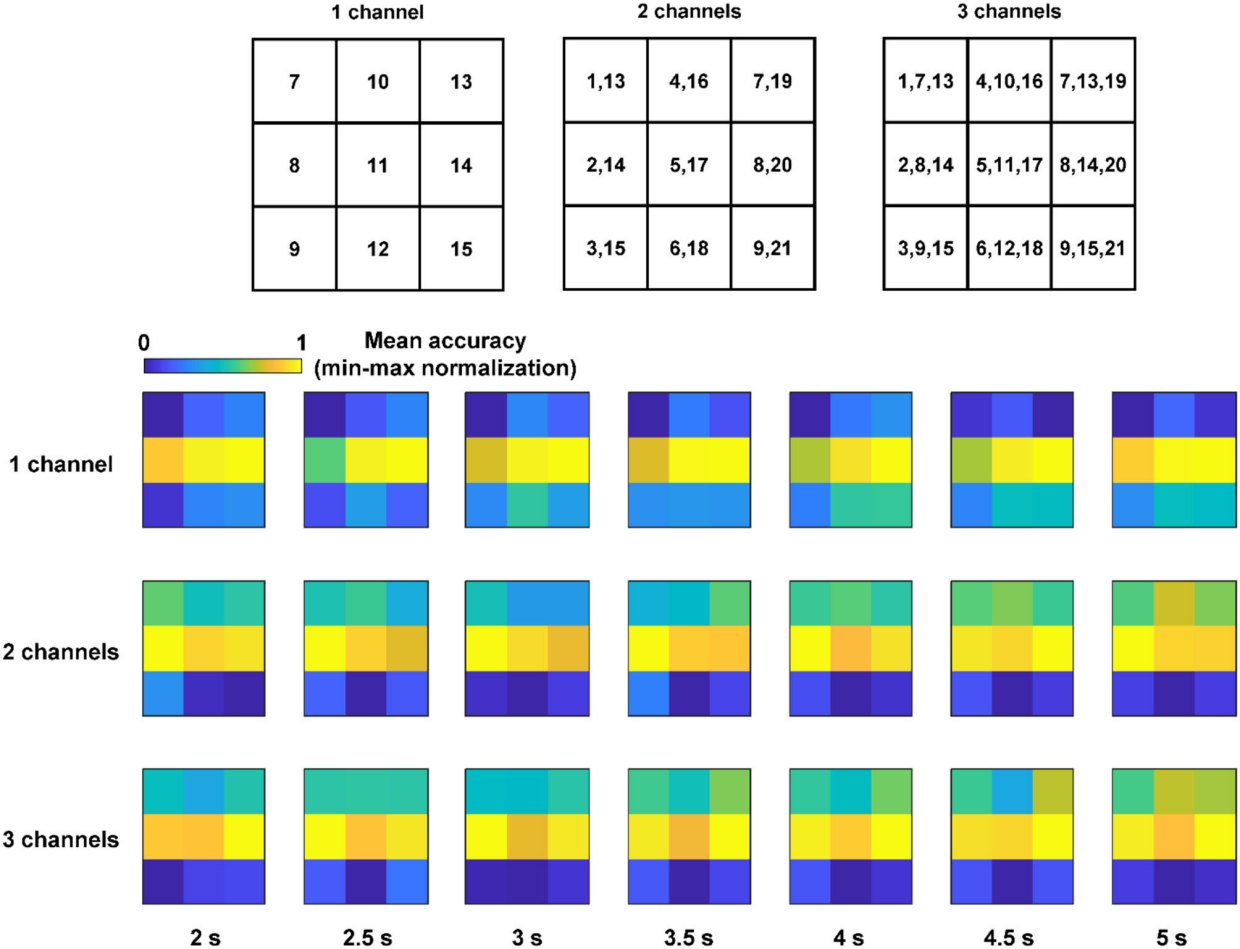
Nine channel combinations corresponding to the cases, in which all the electrodes shift to the same direction and the mean accuracies across the subjects of the nine-channel combinations (accuracies were calculated using EMSI and the nine mean accuracies all the cases with various number of channels and each window length were min–max normalized).

Recently, BCI studies with portable EEG devices have been actively investigated with the aim of enhancing the practical usability of BCIs (Casson, 2019). When using portable EEG devices, it is generally difficult to place the EEG electrodes consistently on the designated locations because most portable devices are not customized to be fitted to the head shape of a specific user. Because the distance between adjacent electrodes in portable EEG devices is usually fixed, we further compared the classification accuracies when all the electrodes moved in the same direction. Figure 4 shows the nine-channel combinations corresponding to the cases in which all the electrodes shifted in the same direction, where the numbers in each element represent the EEG channel numbers. The three rows with colored matrices show the mean classification accuracies across the subjects for the nine-channel combinations with respect to different window sizes. The accuracies were calculated using EMSI, and the nine mean accuracies were min-max normalized. Notably, it could be seen from the figures that horizontal electrode shifts resulted in a relatively smaller decrease in the classification accuracy than vertical electrode shifts.

One of the limitations of our study is that we investigated the performance robustness to a slight electrode shift only for training-free SSVEP detection algorithms. According to a previous study (Zerafa et al., 2018), algorithms detecting SSVEP can be categorized based on training requirements into three categories: training-free, subject-specific, and subject-independent. Training-free algorithms are more practical than the ones included in the other two categories because they do not require any training data for SSVEP classification. However, classification algorithms using training data generally outperform the training-free algorithms because the inter-subject variability of the EEG activity is reflected in the classification. Algorithms belonging to the subject-independent category are expected to be a satisfactory compromise between performance and training effort. Therefore, evaluating the RES of various subject-independent algorithms would be one of the promising topics that we plan to pursue in future studies. Recently, SSVEP-based BCIs employing high-frequency bands have been actively studied because they could effectively reduce visual fatigue, compared to those employing low- and mid-frequency bands (Liang et al., 2019; Hsu et al., 2020). Despite its improved usability, however, its performance was not as high as that of the low- and mid-frequency SSVEP-based BCIs. Therefore, it would be a promising topic to investigate the influence of slight electrode displacement on the performance of high-frequency SSVEP-based BCIs.

## Conclusion

In this study, we investigated the changes in the performance of SSVEP-based BCIs by minor electrode shifts to discover the optimal conditions that minimize the variations in classification accuracy while maintaining high classification accuracy. The performance robustness of SSVEP-based BCIs to the electrode shift with respect to the numbers of channels and classification algorithms was quantitatively investigated using two measures (ACA and RES). Our results suggested that the use of multichannel electrode configurations and employment of the EMSI algorithm could make the performance of SSVEP-based BCIs robust to the electrode shift from the optimal electrode locations. To the best of our knowledge, this was the first study that quantitatively evaluated the influence of electrode shift on the performance of SSVEP-based BCIs.

Recently, commercial wearable EEG devices that can record SSVEP responses in a more convenient manner have been released to the market. The representative examples include NextMind™ (https://www.next-mind.com) and BrainBit (https://www.brainbit.com), both of which employ dry electrodes and elastic headbands. Therefore, investigation on the robustness of BCI performance to the minor electrode shift for commercial wearable EEG devices would be necessary to assess the practicality of these devices. Additionally, it would be an interesting future topic to investigate optimal electrode configurations that are most robust to electrode shift in SSVEP-based BCIs.

## Data Availability Statement

The preprocessed EEG datasets are available at the following link: https://figshare.com/s/cd46e1531d67f3023fa6.

## Ethics Statement

The studies involving human participants were reviewed and approved by Institutional Review Board (IRB) of Hanyang University. The patients/participants provided their written informed consent to participate in this study.

## Author Contributions

HK: conceptualization, data curation, formal analysis, investigation, methodology, validation, visualization, and writing-original draft preparation. C-HI: conceptualization, investigation, supervision, writing-review, and editing. All the authors have reviewed and approved the final manuscript.

## Funding

This work was supported in part by the National Research Foundation of Korea (NRF) (Grant No. 2019R1A2C2086593) and in part by the Institute of Information & Communications Technology Planning & Evaluation (IITP) grant funded by the Korean government (MIST) (No. 2017-0-00432).

## Conflict of Interest

The authors declare that the research was conducted in the absence of any commercial or financial relationships that could be construed as a potential conflict of interest.

## Publisher's Note

All claims expressed in this article are solely those of the authors and do not necessarily represent those of their affiliated organizations, or those of the publisher, the editors and the reviewers. Any product that may be evaluated in this article, or claim that may be made by its manufacturer, is not guaranteed or endorsed by the publisher.
