# Supplementary material for: Influence of the Number of Channels and Classification Algorithm on the Performance Robustness to Electrode Shift in Steady-State Visual Evoked Potential-Based Brain-Computer Interfaces
Source: Front Neuroinform. 2021 Oct 22;15:750839. doi: 10.3389/fninf.2021.750839 (PMC8569408; doi:10.3389/fninf.2021.750839)
Supplement: Supplementary file 1 [file Data_Sheet_1.PDF]

Supplementary Material

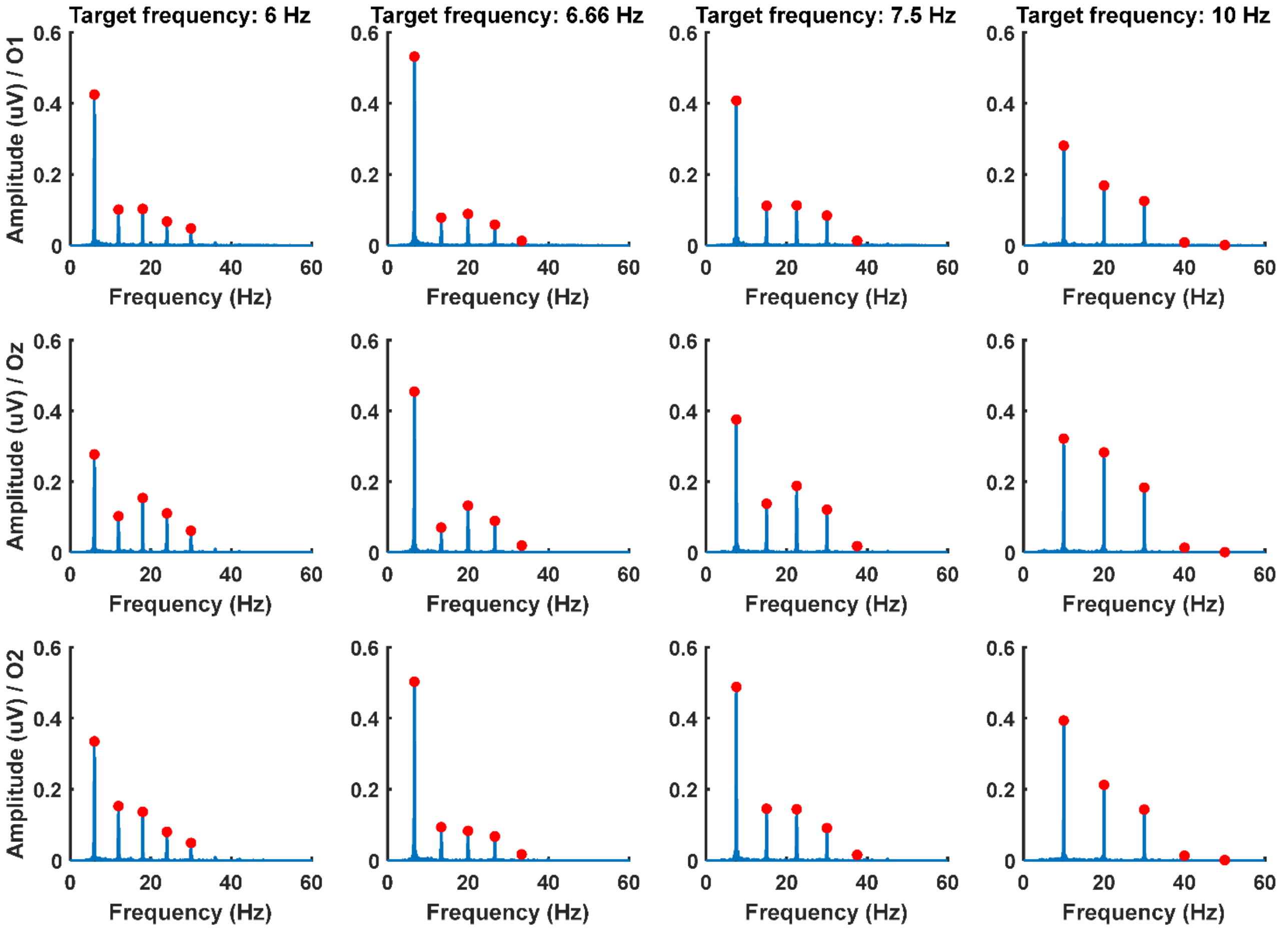

Supplementary Figure 1. Average amplitude spectrum averaged across the 21 subjects and 25 trials for four stimuli frequencies at O1, Oz, and O2 channels. Red circles indicate the target frequencies and their harmonics.

Supplementary Table 1. Mean and standard deviations of the average classification accuracy across channel combination (ACA) across the subjects with respect to the SSVEP classification algorithm, number of channels, and window length.

**Algorithm**	**Number of channels**	**Window length (s)**						
		**2**	**2.5**	**3**	**3.5**	**4**	**4.5**	**5**
CCA	1	0.534 (0.114)	0.595 (0.135)	0.649 (0.136)	0.686 (0.135)	0.718 (0.135)	0.739 (0.134)	0.767 (0.126)
	2	0.549 (0.102)	0.605 (0.109)	0.654 (0.111)	0.699 (0.112)	0.734 (0.110)	0.763 (0.108)	0.792 (0.105)
	3	0.573 (0.116)	0.637 (0.122)	0.688 (0.125)	0.734 (0.122)	0.769 (0.117)	0.798 (0.113)	0.825 (0.108)
ECCA	1	0.536 (0.113)	0.599 (0.133)	0.653 (0.137)	0.690 (0.135)	0.723 (0.134)	0.742 (0.132)	0.768 (0.128)
	2	0.555 (0.106)	0.614 (0.112)	0.664 (0.115)	0.706 (0.116)	0.742 (0.111)	0.772 (0.109)	0.801 (0.104)
	3	0.582 (0.120)	0.649 (0.124)	0.701 (0.127)	0.745 (0.124)	0.780 (0.118)	0.809 (0.114)	0.835 (0.107)
FBCCA	1	0.525 (0.132)	0.592 (0.144)	0.643 (0.151)	0.675 (0.153)	0.708 (0.152)	0.733 (0.150)	0.760 (0.146)
	2	0.549 (0.117)	0.609 (0.128)	0.656 (0.135)	0.697 (0.138'	0.733 (0.135)	0.764 (0.128)	0.794 (0.122)
	3	0.594 (0.135)	0.658 (0.140)	0.709 (0.143)	0.749 (0.143)	0.783 (0.136)	0.813 (0.127)	0.837 (0.118)
MSI	1	0.534 (0.114)	0.594 (0.134)	0.648 (0.135)	0.687 (0.136)	0.718 (0.136)	0.738 (0.133)	0.768 (0.127)
	2	0.571 (0.104)	0.631 (0.115)	0.683 (0.118)	0.721 (0.121)	0.759 (0.114)	0.784 (0.110)	0.811 (0.107)
	3	0.604 (0.111)	0.669 (0.120)	0.724 (0.121)	0.764 (0.120)	0.798 (0.112)	0.823 (0.108)	0.849 (0.104)
EMSI	1	0.541 (0.114)	0.600 (0.134)	0.656 (0.136)	0.693 (0.138)	0.725 (0.135)	0.746 (0.131)	0.773 (0.126)
	2	0.576 (0.105)	0.638 (0.117)	0.690 (0.122)	0.729 (0.123)	0.766 (0.115)	0.792 (0.111)	0.818 (0.107)
	3	0.610 (0.113)	0.676 (0.123)	0.731 (0.125)	0.772 (0.122)	0.806 (0.114)	0.830 (0.110)	0.855 (0.104)

Values denote average (standard deviation) of the ACA across 21 subjects.

Supplementary Table 2. Mean and standard deviations of the robustness against the electrode shift (RES) across the subjects with respect to the SSVEP classification algorithm, number of channels, and window length.

Algorithm	Number of channels	Window length (s)						
		2	2.5	3	3.5	4	4.5	5
CCA	1	0.875 (0.050)	0.880 (0.058)	0.887 (0.055)	0.895 (0.052)	0.900 (0.053)	0.907 (0.053)	0.915 (0.047)
	2	0.869 (0.034)	0.872 (0.037)	0.880 (0.032)	0.890 (0.031)	0.898 (0.033)	0.904 (0.033)	0.913 (0.033)
	3	0.868 (0.025)	0.875 (0.030)	0.884 (0.031)	0.895 (0.032)	0.904 (0.032)	0.910 (0.032)	0.919 (0.033)
ECCA	1	0.875 (0.050)	0.879 (0.057)	0.887 (0.055)	0.893 (0.053)	0.898 (0.053)	0.905 (0.053)	0.915 (0.048)
	2	0.871 (0.034)	0.875 (0.038)	0.882 (0.034)	0.891 (0.032)	0.900 (0.034)	0.907 (0.033)	0.915 (0.034)
	3	0.870 (0.024)	0.878 (0.030)	0.888 (0.031)	0.897 (0.033)	0.907 (0.033)	0.913 (0.034)	0.922 (0.034)
FBCCA	1	0.874 (0.056)	0.878 (0.051)	0.887 (0.051)	0.890 (0.048)	0.897 (0.055)	0.909 (0.055)	0.916 (0.049)
	2	0.860 (0.027)	0.866 (0.032)	0.875 (0.034)	0.885 (0.036)	0.893 (0.035)	0.901 (0.036)	0.910 (0.039)
	3	0.866 (0.024)	0.875 (0.033)	0.884 (0.037)	0.894 (0.039)	0.905 (0.039)	0.913 (0.039)	0.923 (0.038)
MSI	1	0.873 (0.050)	0.880 (0.058)	0.889 (0.056)	0.895 (0.052)	0.899 (0.054)	0.908 (0.053)	0.915 (0.047)
	2	0.886 (0.036)	0.892 (0.038)	0.902 (0.034)	0.907 (0.036)	0.913 (0.035)	0.921 (0.035)	0.927 (0.033)
	3	0.901 (0.031)	0.908 (0.034)	0.919 (0.029)	0.923 (0.033)	0.931 (0.031)	0.936 (0.030)	0.942 (0.030)
EMSI	1	0.874 (0.046)	0.880 (0.055)	0.890 (0.055)	0.899 (0.050)	0.902 (0.052)	0.909 (0.052)	0.919 (0.045)
	2	0.888 (0.035)	0.894 (0.037)	0.905 (0.034)	0.909 (0.036)	0.915 (0.035)	0.923 (0.033)	0.930 (0.031)
	3	0.903 (0.030)	0.910 (0.033)	0.922 (0.027)	0.926 (0.033)	0.934 (0.030)	0.939 (0.028)	0.945 (0.028)

Values denote average (standard deviation) of the RES across 21 subjects.
